# AST-120, an Oral Carbon Absorbent, Protects against the Progression of Atherosclerosis in a Mouse Chronic Renal Failure Model by Preserving sFlt-1 Expression Levels

**DOI:** 10.1038/s41598-019-51292-9

**Published:** 2019-10-30

**Authors:** Yasuki Nakada, Kenji Onoue, Tomoya Nakano, Satomi Ishihara, Takuya Kumazawa, Hitoshi Nakagawa, Tomoya Ueda, Taku Nishida, Tsunenari Soeda, Satoshi Okayama, Makoto Watanabe, Rika Kawakami, Yoshihiko Saito

**Affiliations:** Department of Cardiovascular Medicine, Nara Medical University, Kashihara, Nara 634-8522 Japan

**Keywords:** Calcification, Atherosclerosis

## Abstract

Soluble Flt-1 (sFlt-1), an endogenous antagonist of the proatherogenic cytokine placental growth factor, is decreased in chronic kidney disease (CKD), leading to atherosclerotic progression. In this study, we investigated the effect of AST-120, an oral carbon adsorbent which can remove uremic toxins, on sFlt-1 expression levels and atherosclerosis progression. Atherosclerotic apolipoprotein E-deficient mice underwent a 5/6 nephrectomy (5/6 NR) or a sham operation (sham) at 8 weeks of age and were then treated or not with oral AST-120 for 12 weeks. sFlt-1 expression levels and the degree of atherosclerosis were assessed at 22 weeks of age in each of the four groups (sham; n = 7, 5/6 NR; n = 10, sham + AST-120: n = 8, 5/6 NR + AST-120; n = 8). The expression levels of sFlt-1 mRNA in the kidney were significantly lower in the 5/6 NR group than in the sham group, but AST-120 treatment prevented this decrease in sFlt-1 levels. Similarly, the atherosclerotic plaque area of the thoracoabdominal aorta was significantly larger in the 5/6 NR group than in the sham group, and AST-120 treatment prevented this increase in atherosclerosis. AST-120 could, therefore, be used as a therapeutic to treat atherosclerosis in patients with CKD.

## Introduction

Previous epidemiological studies have revealed that patients with chronic kidney disease (CKD) are at a markedly higher risk for cardiovascular morbidity and mortality^1–7^. However, the molecular mechanisms connecting renal dysfunction with the progression of atherosclerosis and the development of cardiovascular diseases have not been fully clarified. Recently, the signalling pathway initiated by placental growth factor (PlGF), a member of the vascular endothelial growth factor family, through its receptor, fms-like tyrosine kinase-1 (Flt-1), has been speculated to be involved in the pathogenesis of atherosclerotic diseases^8–11^. PlGF selectively binds to Flt-1 and accelerates atherosclerotic processes by enhancing intramural angiogenesis and monocyte recruitment. Interestingly, a soluble isoform of Flt-1, which is produced by alternative splicing of the full-length Flt-1 mRNA and consists of only the extracellular domains, can act as an endogenous inhibitor of PlGF, that is, it acts as a decoy receptor^12–14^. We have demonstrated that a reduction in the circulating levels of sFlt-1 is associated with the renal dysfunction that accompanies the worsening of atherosclerosis^15^. We have also reported that serum from patients with CKD induces endothelial damage and suppresses sFlt-1 production^16^. Although the factors regulating sFlt-1 expression have not been clearly elucidated, we hypothesised that uremic toxins are involved in the reduction of sFlt-1 in patients with CKD.

AST-120, an oral charcoal adsorbent, is widely utilised as a therapeutic agent to depress circulating uremic toxins in patients with CKD^17,18^. In a retrospective study, AST-120 contributed to a reduction in the prevalence of cardiac events and mortality in patients with CKD^19^. Since no drugs have been developed to regulate the production of sFlt-1 so far, in this study, we investigated the effect of AST-120 on sFlt-1 expression levels and the impact on atherosclerotic progression.

## Results

### The expression levels of sFlt-1 are decreased by uremic toxins in HUAECs

**Figure 1:**
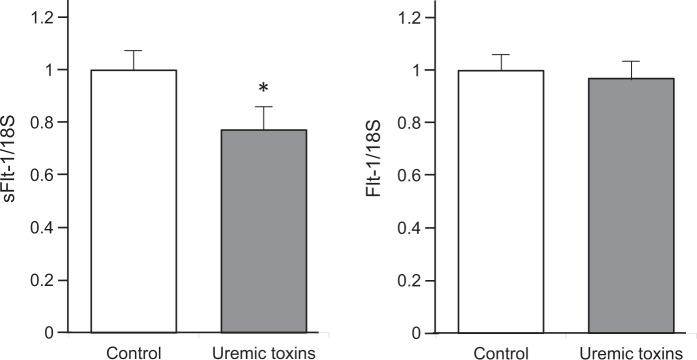
Uremic toxins reduce the levels of soluble fms–like tyrosine kinase-1 (sFlt-1) expression in endothelial cells. Human umbilical artery endothelial cells (HUAECs) were cultured with uremic toxins, and the levels of the mRNAs encoding sFlt-1 and Flt-1 were measured by real-time reverse transcription–polymerase chain reaction (RT-PCR) after 24 h; n = 4. *p < 0.05 versus control. Data are mean ± SEM. Uremic toxins; indoxyl sulphate 100 μg/mL and p-Cresol 50 μg/mL.

To investigate the mechanisms underlying the reduction in sFlt-1 expression in patients with CKD, cultured human umbilical artery endothelial cells (HUAECs) were incubated with uremic toxins. The expression levels of sFlt-1 mRNA were significantly decreased in cultures with uremic toxins compared with those in control cultures (n = 4, p  < 0.05). There were no significant differences in the expression levels of Flt-1 (n = 4, p = 0.8041) (Fig. 1).

### Effect of AST-120 on physical and biochemical parameters in a CKD animal model

We established a mouse model of chronic kidney disease (CKD) using male ApoE-deficient mice who had undergone a 5/6 nephrectomy (5/6NR) and used this model to assess the effect of AST-120 on physical and biochemical parameters. We made four different treatment groups which consist of sham operation with no treatment (sham, n = 7), 5/6NR with no treatment (5/6NR, n = 8), sham operation with AST-120 treatment (sham + AST-120, n = 10), and 5/6NR with AST-120 treatment (5/6NR + AST-120, n = 8). There were no significant differences in body weight, heart rate, blood pressure, and the heart/body weight ratio among the four experimental groups at the end of the study (Table 1). Both serum indoxyl sulphate levels and blood urea nitrogen (BUN) levels were significantly higher in the 5/6NR mice than in the sham mice without AST-120 treatment (p < 0.001 for both). In the 5/6NR + AST-120 mice, the serum p-cresyl sulphate levels were significantly lower (p < 0.01) and the serum indoxyl sulphate levels tended to be lower (p = 0.08) than in the 5/6NR mice without AST-120 treatment. AST-120 had no significant effect on serum BUN levels. In echocardiography at the end of the study, there was no significant difference in wall thickness and left ventricular systolic function among the four experimental groups (Supplementary Table S1).

**Table 1:** Hemodynamics and Biological Parameters in Control and 5/6-Nephrectomized ApoE-Deficient Mice at the End of the Study.

	AST120 (−)		AST120 (+)	
	sham (n = 7)	5/6NR (n = 8)	sham (n = 10)	5/6NR (n = 8)
Body weight (g)	26.8 ± 0.7	26.5 ± 0.5	29.1 ± 1.2	26.0 ± 0.6
Heart rate (beats/min)	628 ± 25	660 ± 15	579 ± 17	621 ± 18
Systolic blood pressure (mmHg)	98 ± 2	96 ± 3	96 ± 3	100 ± 3
Diastolic blood pressure (mmHg)	53 ± 2	58 ± 2	51 ± 2	51 ± 3
Heart/body weight (mg/g)	3.8 ± 0.2	3.9 ± 0.1	3.7 ± 0.2	4.1 ± 0.2
Blood urea nitrogen (mg/dl)	26.6 ± 1.4	44.5 ± 1.9*	25.7 ± 1.2	48.7 ± 2.5
Indoxyl sulfate (mg/dl)	0.17 ± 0.02	0.33 ± 0.03*	0.17 ± 0.02	0.24 ± 0.04
p-cresyl sulphate (mg/dl)	0.06 ± 0.01	0.07 ± 0.02	0.02 ± 0.01	0.03 ± 0.01^†^

Data are mean ± SEM. *p < 0.001 vs sham AST120(−). ^†^p < 0.01 vs 5/6NR AST120(−).

### AST-120 preserves the expression of sFlt-1 in the CKD animal model

**Figure 2:**
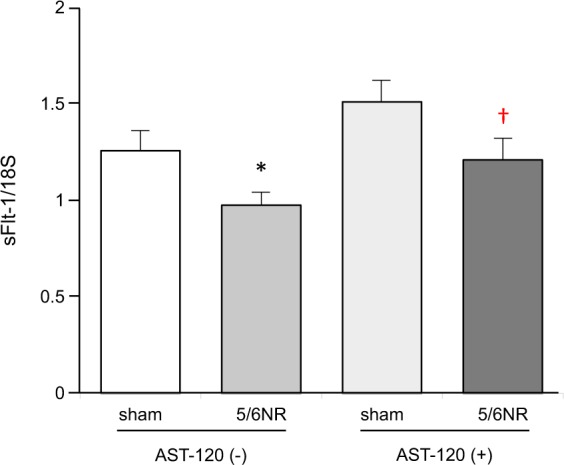
The effect of AST-120 on the renal expression of sFlt-1 mRNA. Renal expression of sFlt-1 was measured in sham operated mice without AST-120 treatment (sham, n = 7), 5/6 nephrectomized mice without AST-120 treatment (5/6NR, n = 8), sham operated mice with AST-120 (sham + AST-120, n = 10), and 5/6NR mice with AST-120 (5/6NR + AST-120, n = 8), respectively. *p < 0.05 vs. sham without AST-120. ^†^p < 0.05 vs. 5/6NR without AST-120. Data are mean ± SEM. 5/6NR indicates 5/6 nephrectomy.

At the end of the study, we harvested the kidney from each mouse in the study and measured the expression levels of sFlt-1 mRNA using quantitative RT-PCR. As shown in Fig. 2, the expression level of sFlt-1 mRNA were significantly lower in the 5/6 NR mice than in the sham operated mice (0.97 ± 0.06 versus 1.26 ± 0.08 arbitrary units normalized to 18 S mRNA; p < 0.05). The extent of this reduction in sFlt-1 levels was reduced in the 5/6 NR + AST-120 mice compared to the 5/6 NR mice (0.97 ± 0.06 versus 1.21 ± 0.09 arbitrary units normalized to 18 S mRNA; p < 0.05).

### AST-120 suppresses the progression of atherosclerosis in a CKD animal model

**Figure 3:**
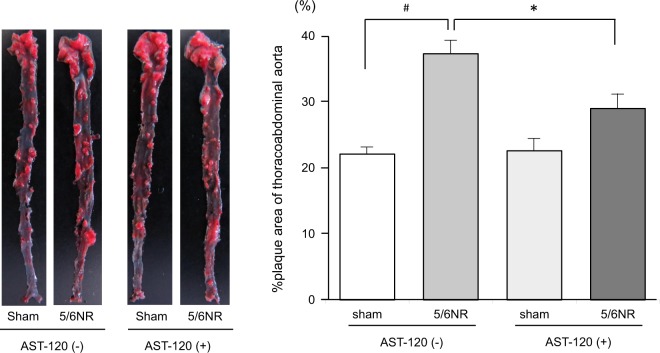
Atherosclerotic plaque formation in the thoracoabdominal aorta. Representative pictures of an opened thoracoabdominal aorta stained with Oil Red O are presented on the left. The plaque areas of the thoracoabdominal aorta in sham operated mice without AST-120 treatment (sham, n = 7), 5/6NR mice without AST-120 treatment (5/6NR, n = 8), sham operated mice with AST-120 (sham + AST-120, n = 10), and 5/6NR mice with AST-120 (5/6NR + AST-120, n = 8) are shown on the right. ^#^p < 0.001, *p < 0.05. Data are mean ± SEM. 5/6NR indicates 5/6 nephrectomy.

The atherosclerotic plaque area of the thoracoabdominal aorta was significantly larger in the 5/6 NR group than in the sham operated group (37.3 ± 2.2% vs 22.1 ± 1.0%, p < 0.001). The degree of atherosclerosis was significantly diminished in the 5/6 NR + AST-120 group compared to the 5/6 NR group (29.0 ± 2.3% vs 37.3 ± 2.2%, p < 0.05) (Fig. 3). On the other hand, we found no significant difference in the degree of atherosclerosis in sham group with or without AST-120.

### The expression levels of sFlt-1 mRNA are negatively correlated with plaque area

**Figure 4:**
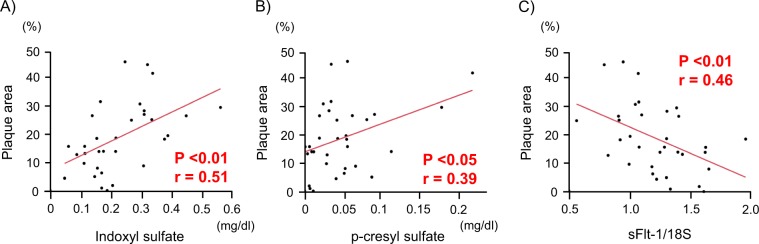
Correlations between the plaque area and each parameter. (**A**) A significant positive correlation was found between the plasma levels of indoxyl sulphate and the atherosclerotic plaque area of the thoracoabdominal aorta. n = 33; r = 0.51; p < 0.01. (**B**) A significant positive correlation was found between plasma levels of p-cresyl sulphate and the atherosclerotic plaque area of the thoracoabdominal aorta. n = 33; r = 0.39; p < 0.05. (**C**) A significant negative correlation was found between the expression levels of sFlt-1 mRNA and the atherosclerotic plaque area of the thoracoabdominal aorta. n = 33; r = −0.46; p < 0.01.

Both the plasma levels of indoxyl sulphate and p-cresyl sulphate, which are uremic toxins, showed a significant positive correlation with the atherosclerotic plaque area of the thoracoabdominal aorta (r = 0.51, p < 0.01: Fig. 4A; r = 0.39, p < 0.05: Fig. 4B, respectively). Additionally, there was a significant negative correlation between the expression levels of sFlt-1 mRNA and the atherosclerotic plaque area of the thoracoabdominal aorta (r = 0.46, p < 0.01) (Fig. 4C).

## Discussion

The present study is the first to demonstrate the ability of an agent to preserve the expression of sFlt-1, a protein which contributes to the inhibition of atherosclerosis. This study suggests the possibility that uremic toxins present in patients with CKD lead to a decrease in sFlt-1 expression and that AST-120 can suppress the progression of atherosclerosis by removal of these uremic toxins.

The pathophysiology of atherosclerosis in patients with CKD arises not from a single factor but from multiple factors, and it is thought that both risk factors that induce CKD such as diabetes or hypertension and mechanisms that are further exacerbated in CKD, such as chronic inflammation derived from activation of the PlGF/Flt-1 pathway, vascular endothelial damage due to the renin-angiotensin system, or sympathetic nerve stimulation and the dysregulation of calcium and phosphorus, are involved^20,21^. Therefore, in patients with CKD, it is not likely to be possible to completely suppress the progression of atherosclerosis simply by maintaining sFlt-1 expression levels, nevertheless sFlt-1 could be considered to be a therapeutic target.

We have reported that activation of the PlGF/Flt-1 pathway caused by a reduction in sFlt-1 expression levels leads to atherosclerosis in patients with CKD. Onoue *et al*. showed that plasma sFlt-1 levels and renal sFlt-1 mRNA expression levels were positively correlated with the estimated glomerular filtration rate in humans. These reductions in circulating sFlt-1 and renal sFlt-1 mRNA levels were also found in 5/6 NR mice that had experimentally induced renal dysfunction^15^. In addition, this previous study showed that the plaque areas in the thoracoabdominal aorta were significantly larger in 5/6 NR ApoE-deficient mice treated with PBS than in control ApoE-deficient mice, and that replacement therapy with recombinant sFlt-1 significantly reduced plaque formation. Matsui *et al*. also reported that the total aortic plaque area in sFlt-1^−/−^/ApoE^−/−^ mice was significantly larger than that in sFlt-1^+/+^/ApoE^−/−^ mice^16^. The factors that regulate sFlt-1 expression levels are not known. In this regard, we previously reported that the addition of serum from patients with CKD to vascular endothelial cell cultures causes a reduction in sFlt-1 expression levels, and therefore, in this study, we focused on uremic toxins that are elevated in patients with CKD. For this purpose, indoxyl sulphate and p-Cresol were used as uremic toxins in the *in vitro* experiments. It has already been reported that indoxyl sulphate and p-Cresol can affect endothelial proliferation and wound repair^22^, and that these uremic toxins are associated with increased vascular stiffness, calcification, and mortality in patients with CKD^23,24^. In this study, we used indoxyl sulphate and p-Cresol at concentrations of 100 μg/mL and 50 μg/mL, respectively, but these concentrations are likely to be higher than those seen in patients with CKD or in *in vivo* CKD models. Further studies are required to elucidate what levels of uremic toxins lead to an increased risk of atherosclerosis. Furthermore, the mechanisms underlying the reduced sFlt-1 production from the endothelial cells by the addition of uremic toxins is unclear. In this study, under the above conditions, the addition of uremic toxins reduced sFlt-1 expression in cultured HUAECs. In our previous study described above, the addition of serum from patients with CKD5 and CKD5D upregulated biomarkers for endothelial injury, i.e., selectin, intercellular adhesion molecule 1, and vascular cell adhesion protein 1^16^. Given that ROS production is augmented with uremic toxins^25^, it is possible that ROS causes endothelial injury and consequently not only inhibits sFlt-1 production but also decreases its storage on the endothelial cells. However, further studies are necessary to clarify the involvement of uremic toxins such as indoxyl sulphate, p-Cresol, and other unknown substances that could reduce sFlt-1 production by various mechanisms.

In this study, AST-120, an oral charcoal adsorbent, was used in the *in vivo* experiments. In a large randomized trial, AST-120 failed to demonstrate beneficial effects at the primary endpoints including such as dialysis initiation and kidney transplantation^26^. However, in this trial, the eGFR change from baseline in AST-120 group was significantly smaller than that of in the placebo group (p = 0.04). Schulman *et al*. also reported that, in post hoc analysis in this trial population, treatment with AST-120 suspended the time to the primary endpoints stated above in patients with progressive CKD receiving standard therapy^27^. Additionally, several papers reported that AST-120 reduces circulating and tissue uremic toxins and delays the progression of experimental and human CKD, at least partially^18,28–31^. In the present study, we used a 5/6 nephrectomy (5/6 NR) in Apo E-deficient mice to cause renal dysfunction, with the result that plasma BUN levels increased to high levels as previously reported^32^. AST-120 treatment did not change the plasma BUN levels over the duration of the study, as previously reported^33,34^; however, p-cresyl sulphate levels were significantly decreased (p < 0.01) and indoxyl sulphate levels also tended to be decreased (p = 0.08) (Table 1). AST-120 also suppressed atherosclerosis in the 5/6NR mouse model, as shown by reductions in the atherosclerotic plaque area of the thoracoabdominal aorta. We suppose that AST-120 reduced uremic toxins that had been elevated in CKD, which may have led to the suppression of the progression of atherosclerosis. With regard to the possibility that AST-120 would suppress atherosclerosis independently of renal function, we found no significant difference in the degree of atherosclerosis in sham group with or without AST-120 (Fig. 3). This result suggests that AST-120 does not have sufficient effects on atherosclerosis in the absence of CKD and the reduction in uremic toxins levels caused by CKD could lead to the suppression of atherosclerosis. While removal of the two above-mentioned uremic toxins may have been effective, other uremic toxins are also involved in CKD, and further investigation is required to elucidate the association between these uremic toxins and atherosclerosis.

The present study had following limitations. First, the effects of AST-120 in the continuously downregulated condition of sFlt-1 was not evaluated. Second, the mechanism of the effect by AST-120 other than PlGF/Flt-1 pathway was not examined. Several mechanisms for the effects of AST-120 have been reported. Yamamoto *et al*. reported that AST-120 inhibited the progression of atherosclerosis^35^. They demonstrated AST-120 lessens inflammatory mRNA expression levels such as of MCP-1, TNF-α and IL-1β in the aorta. Regarding this, the relationship between PlGF and MCP-1 up-regulation has been reported^36^, and it is possible that the reduced inflammation is associated with the suppression of PlGF/Flt-1 pathway. Other than PlGF/Flt-1 pathway, Nishikawa *et al*. reported AST-120 improved mitochondrial biogenesis of skeletal muscle via reducing oxidative stress^34^. Considering that the relationship between mitochondrial dysfunction and atherosclerosis has been reported^37^, AST-120 may have improved mitochondrial function and led to the suppression of atherosclerosis. In addition, Indoxyl sulfate, one of uremic toxins, has been reported to cause endoplasmic reticulum stress^38^. The correlation between endoplasmic reticulum stress and arteriosclerosis has also been pointed out^31^, thus, improved endoplasmic reticulum stress by removing uremic toxins might be related to the reduction of the degree of atherosclerosis. In conclusion, our study showed that AST-120 can preserve the levels of sFlt-1 and suppress the progression of atherosclerosis through the removal of uremic toxins in ApoE-deficient mice with impaired renal function. These results suggest that AST-120 may be an effective novel therapeutic agent for the treatment of cardiovascular events in patients with CKD.

## Methods

### Experimental Study

#### Cells and culture experiments

Human umbilical artery endothelial cells (HUAECs) were obtained from Lonza (Allendale, NJ, USA). For the purpose of investigating sFlt-1 expression levels, HUAECs were seeded in 6-well dishes at a cell density of 3 × 10^4^ cells per well and incubated with endothelial cell growth medium, and treated with uremic toxins, namely 100 μg/mL indoxyl sulphate and 50 μg/mL p-Cresol. After 24 h of incubation, cells were harvested for the measurement of sFlt-1 expression levels.

#### Mouse model of renal dysfunction

Male ApoE-deficient mice (C57BL/6 background) were purchased from Taconic Farms (Hudson, NY, USA) and maintained in a temperature-controlled room with a 12-hour light/dark cycle and free access to water and standard chow. At 8 weeks of age, the mice were randomly assigned to a 5/6 nephrectomy group (chronic renal failure; removal of one kidney and two thirds of the other kidney) or a control group. The 5/6 nephrectomy (5/6NR) operation was performed as described previously^39–41^. At 12 weeks of age, each of these groups were then further subdivided; sham operation with no treatment (sham, n = 7), 5/6NR with no treatment (5/6NR, n = 8), sham operation with AST-120 (sham + AST-120, n = 10), and 5/6NR with AST-120 (5/6NR + AST-120, n = 8). They were maintained on a Western diet (16.5% fat, 1.25% cholesterol, 0.5% sodium cholate) or Western diet with AST-120 from 12 to 22 weeks old. AST-120 was administered as pulverised chow at 8% (w/w), as previously reported^42^. All animal experiments performed for this study were approved by The Animal Care and Use Committee at Nara Medical University (experimental number 110-3) and all methods were performed based on the Policy on the Care and Use of Laboratory Animals, Nara Medical University.

#### Blood and mRNA analysis

Total cholesterol and triglyceride levels were assayed with enzymatic kits (Wako Pure Chemical Industries, Osaka, Japan). Measurements of indoxyl sulphate and p-cresyl sulphate were performed as described previously^43^. Plasma BUN levels were measured by urease-glutamate dehydrogenase method using UniCel DxC 600 (Beckman Coulter, CA, USA). We extracted mRNA from frozen renal samples and synthesized cDNA using standard protocols. The relative levels of sFlt-1 mRNA were then determined by real-time reverse transcription polymerase chain reaction (RT-PCR) with gene specific primers as follows; mouse: 5′-CTCTAGAAGACTCGGGCACC-3′ (forward) and 5′-GAGCGTTTCCTCTGGGCCTG-3′ (reverse); human: 5′-CCCTGCAAGATTCAGGCACC-3′ (forward) and 5′-GAGCATCTCCTCCGAGCCTG-3′ (reverse). The expression levels of full-length Flt-1 mRNA were measured using RT-PCR with gene specific primers as follows; mouse: 5′-CTCTAGAAGACTCGGGCACC-3′ (forward) and 5′-GAGGCGCGGGGACACCTCTAG-3′ (reverse); human: 5′-CCCTGCAAGATTCAGGCACC-3′ (forward) and 5′-GGCTCGGGGACACCATTAGC-3′ (reverse). The levels of sFlt-1 and Flt-1 mRNAs were normalized to those of 18 S mRNA.

#### Measurement of atherosclerotic lesions and histological examination

To quantify the atherosclerotic plaque formation, atherosclerotic lesions within the thoracoabdominal aorta were stained with Oil Red O. Plaque areas were traced by two independent examiners who were blinded to the specimens’ background and were measured with ImageJ version 1.45 software (http://rsb.info.nih.gov/ij/).

#### Echocardiography

Transthoracic echocardiography studies were performed, in animal anesthetized with isoflurane (1 to 2%), using a Prospect (S-Sharp Corporation, Taipei, Taiwan) with a 40 MHz transducer at the end of the study. Warmed echo gel was placed between the probe and shaved chest. B-mode and M-mode images of the heart were obtained from parasternal short axis. LV fractional shortening was calculated using M-mode imaging.

### Statistical analysis

Continuous data are expressed as the mean ± standard error unless otherwise indicated. A Student’s *t*-test was used for the comparison of continuous variables between the two groups. A linear regression analysis was performed to assess correlations between two continuous variables. P < 0.05 was considered as statistically significant. All statistics were calculated with JMP software for Windows version 13 (SAS Institute, Cary, NC, USA).

## Supplementary information

Supplementary Table S1
