# Supplementary material for: AST-120, an Oral Carbon Absorbent, Protects against the Progression of Atherosclerosis in a Mouse Chronic Renal Failure Model by Preserving sFlt-1 Expression Levels
Source: Sci Rep. 2019 Oct 30;9:15571. doi: 10.1038/s41598-019-51292-9 (PMC6821698; doi:10.1038/s41598-019-51292-9)
Supplement: Supplementary file 1 — Supplementary Table S1 [file 41598_2019_51292_MOESM1_ESM.docx]

**AST-120, an Oral Carbon Absorbent, Protects against the Progression of Atherosclerosis in a Mouse Chronic Renal Failure Model by Preserving sFlt-1 Expression Levels**

Yasuki Nakada, Kenji Onoue*, Tomoya Nakano, Satomi Ishihara, Takuya Kumazawa, Hitoshi Nakagawa, Tomoya Ueda, Taku Nishida, Tsunenari Soeda, Satoshi Okayama, Makoto Watanabe, Rika Kawakami, Yoshihiko Saito

Department of Cardiovascular Medicine, Nara Medical University, Kashihara, Nara 634-8522, Japan

*Address for correspondence: Kenji Onoue

Nara Medical University, 840, Shijo-cho

Kashihara, Nara 634-8522, Japan

Tel: +81-744-22-3051

Fax: +81-744-22-9726

Email: konoue@naramed-u.ac.jp

**Supplementary Table S1. Echocardiographic Parameters in Control and 5/6-Nephrectomized ApoE-Deficient Mice at the End of the Study**

|  | **AST120 (-)** | | | | | |  | **AST120 (+)** | | | | | |
| --- | --- | --- | --- | --- | --- | --- | --- | --- | --- | --- | --- | --- | --- |
|  | **sham (n=5)** | | | **5/6NR (n=7)** | | |  | **sham (n=6)** | | | **5/6NR (n=5)** | | |
| IVSd (mm) | 0.72 | ± | 0.02 | 0.74 | ± | 0.01 |  | 0.71 | ± | 0.02 | 0.74 | ± | 0.01 |
| LVPWd (mm) | 0.66 | ± | 0.01 | 0.69 | ± | 0.02 |  | 0.67 | ± | 0.01 | 0.66 | ± | 0.01 |
| LVDd (mm) | 3.38 | ± | 0.08 | 3.08 | ± | 0.10 |  | 3.43 | ± | 0.17 | 3.46 | ± | 0.22 |
| LVDs (mm) | 2.23 | ± | 0.08 | 2.01 | ± | 0.05 |  | 2.21 | ± | 0.08 | 2.15 | ± | 0.11 |
| Fractional shortning (%) | 34.2 | ± | 0.2 | 34.8 | ± | 0.1 |  | 35.4 | ± | 0.1 | 37.7 | ± | 0.1 |

Data are mean ± SEM. IVSd, interventricular septal wall thickness at end diastole; LVPWd, left ventricular posterior wall thickness at end diastole; LVDd, left ventricular diameter at end diastole; LVDs, left ventricular diameter at end-systole.
